# GM-CSF regulates ILC states and myeloid cell signaling during ulceration in Crohn’s disease

**DOI:** 10.64898/2026.02.03.703526

**Published:** 2026-02-05

**Authors:** Joshua K. Morrison, Ksenija Sabic, Neha Maskey, Sayali Talware, Nai-yun Hsu, Colleen Chasteau, Elizabeth Aslinger, Jake Herb, Shikha Nayar, Rachel Levantovsky, Christopher Tastad, Rachel Moss, Alan Soto, Monica Garcia-barros, Jessy Ntunzwenimana, Mariel Glass, Michelle Bao, Jiayu Zhang, Huajun Han, Jane Stevens, Lorena Tavares, Tin Htwe Thin, Sergey Khaitov, Alexander Greenstein, Rachel Brody, Jaime Chu, Arthur Mortha, Judy H. Cho, Ling-shiang Chuang

**Affiliations:** 1 Department of Pathology, Molecular, and Cell-Based Medicine, Icahn School of Medicine at Mount Sinai, New York, NY, United States.; 2 Hunter College, New York, NY, United States.; 3 Division of Gastroenterology, Icahn School of Medicine at Mount Sinai, New York, NY, United States.; 4 Graduate School of Biomedical Sciences, Icahn School of Medicine at Mount Sinai, New York, NY, United States.; 5 Department of Immunology and Immunotherapy, Icahn School of Medicine at Mount Sinai, New York, NY, United States.; 6 Department of Pediatrics, Icahn School of Medicine at Mount Sinai, New York, NY, United States.; 7 Department of Immunology, University of Toronto, Toronto, Canada.

**Keywords:** Crohn’s Disease, Colony-stimulating Factors, Innate Lymphoid Cells, Ulceration, Spatial Transcriptomics

## Abstract

Macrophage (M-), granulocyte (G-), and granulocyte–macrophage (GM-) colony-stimulating factors (CSFs) regulate myeloid cell function, yet their relative roles during inflammation remain poorly defined. To uncover how CSFs shape spatial immune niches in Crohn’s disease, we performed Xenium single-cell spatial transcriptomics on ileal tissues, revealing cell-type–specific expression and source–target interactions for each CSF. GM-CSF, unlike M-CSF or G-CSF, was locally enriched in ulcerated regions where lymphocytes adjacent to macrophage aggregates signaled through STAT5 phosphorylation. To study functional consequences, we developed a csf2rb^−^/^−^ zebrafish model of intestinal injury. Using this model, we found that loss of GM-CSF signaling exacerbated epithelial damage and inflammation, whereas recombinant human GM-CSF limited injury by restraining ILC1 expansion, sustaining ILC3 maintenance, and promoting IL-22 production. Cross-species single-cell analysis revealed conserved ILC gene modules and GM-CSF–dependent transcriptional networks linking lymphoid and myeloid populations. These findings establish GM-CSF as a critical spatial regulator of myeloid–lymphoid crosstalk and intestinal immune homeostasis in Crohn’s disease.

## Introduction

Colony stimulating factor (CSF) cytokines, including macrophage-CSF (M-CSF/*CSF1*), granulocyte-macrophage-CSF (GM-CSF/*CSF2*), and granulocyte-CSF (G-CSF/*CSF3*) are central regulators of myelopoiesis. While M-CSF and G-CSF are essential for macrophage and neutrophil regulation, respectively, GM-CSF is relatively dispensable during steady-state, apart from supporting alveolar macrophages and tissue dendritic cells^1^. During intestinal inflammation, however, GM-CSF promotes myeloid activation, cell migration, and macrophage renewal, making it a key player in Crohn’s disease (CD) pathogenesis. CD, a chronic inflammatory bowel disease, involves dysregulated myeloid activity^2^, underscoring the importance of dissecting the distinct roles of CSF cytokines in inflammation.

Genome-wide association studies have linked *CSF2RB*, the GM-CSF receptor beta chain, to IBD risk^2,3^. Further, the presence of neutralizing anti-GM-CSF autoantibodies has been associated with increased CD complications^4^. Recent studies suggest GM-CSF, through phosphorylation of STAT5^5^, can induce tolerogenic states in monocytes and dendritic cells, important for maintaining epithelial homeostasis^5,6^. However, conflicting pre-clinical results^7–9^, including mice deficient in GM-CSF or CSF2RB and anti-GM-CSF neutralizing antibody administration, highlight the need for deeper insights into its mechanisms and cellular targets in IBD.

Within the intestine, innate lymphocytes, particularly group 3 innate lymphoid cells (ILC3s), are major GM-CSF producers^6,10^. ILCs orchestrate mucosal immunity through integration of cytokine, stress, and microbial signals, and ILC3s support intestinal epithelial homeostasis via IL-17 and IL-22 production^11^. ILC1, a proinflammatory, interferon-gamma (IFNg) producing subtype, expansion and ILC3 contraction are hallmarks of CD inflammation, with anti-GM-CSF autoantibodies correlating with this imbalance^4,12^, implicating GM-CSF in ILC regulation.

Many genes associated with CD, particularly those with the largest effect sizes, are expressed by myeloid cells and reflect a genetic architecture shaped by host responses to mycobacterial species^2^. Importantly, the zebrafish pathogen, *Mycobacterium marinum*, has provided substantial *in vivo* insight into human mycobacterial pathogenesis^13,14^. To investigate intestinal inflammation, we employed the dextran sodium sulfate (DSS)-induced intestinal injury model in larval zebrafish—a well-established system for studying epithelial damage and innate immune activation. This model leverages the organism’s optical transparency, conserved immune cell lineages and signaling pathways, and, critically, its lack of functional adaptive immunity, which enables direct assessment of innate immune cell–driven processes during mucosal injury and repair healing^15,16^. Moreover, the recent identification of ILC-like cells with transcriptional features analogous to mammalian counterparts further supports the zebrafish as a powerful model for dissecting GM-CSF–dependent innate immune mechanisms^17^.

While single-cell RNA sequencing has advanced our understanding of cytokine signaling in IBD^16,18,19^, it lacks the spatial resolution necessary to contextualize cellular interactions. Given the limited detection of GM-CSF in serum, even during inflammation, and GM-CSF largely being produced by tissue resident cells^4,6^, investigating the local, cell type-specific effects of GM-CSF requires both single-cell and spatial resolution. To answer these questions, we leverage Xenium single-cell spatial transcriptomics tools to interrogate GM-CSF’s cell-type-specific effects within intestinal tissues^20^.

Defective GM-CSF signaling, whether caused by neutralizing anti–GM-CSF autoantibodies^4^ or loss-of-function mutations in *CSF2RB*^5^, has been linked to increased susceptibility to CD and greater risk of disease complications. Defining the cell-type specific and spatial mechanisms by which GM-CSF influences intestinal immunity is therefore essential. By integrating Xenium spatial transcriptomics from paired inflamed and uninvolved CD tissues with functional analyses in a *csf2rb*^*−/−*^ zebrafish model, we uncover a previously unrecognized role for GM-CSF in modulating innate lymphoid cell (ILC) states and intestinal inflammation, providing mechanistic insight into how GM-CSF maintains mucosal immune homeostasis.

## Methods

### Ileal tissue collection and processing

Ileocolic resection samples were examined by a pathologist, with transmural sections taken from inflamed, uninflamed, and stricture regions within an hour. Tissues were fixed in 10% formalin for 48 hours, preserved in 70% ethanol for up to 72 hours, and then embedded in paraffin at Mount Sinai’s Biorepository and Pathology Core.

### Xenium In Situ spatial transcriptomics

FFPE blocks were sectioned (5μm) at Mount Sinai’s Biorepository and Pathology Core per 10x Genomics protocol CG000578. Sections were placed on Xenium slides, dried, deparaffinized, and permeabilized. mRNAs were targeted using specific probes, hybridized overnight at 50°C. After washing, probes were ligated (37°C, 2h), then amplified enzymatically to enhance signal. Background fluorescence was quenched, and sections were imaged using the Xenium Analyzer, which performed automated imaging, liquid handling, and analysis. Z-stacks were acquired with a 0.75 μm step size.

### Xenium data processing

Cells were filtered and gene expression data normalized according to Xenium standard preprocessing procedures using the scanpy python package and python version 3.10.4. Filtering parameters included (a) the minimum number of genes expressed per cell and transcript counts per cell (for cell filtering) and (b) the minimum number of transcripts for each gene and number of cells in which genes were expressed (for gene filtering, though no genes were removed as all passed the filtering parameters). The gene expression matrix was total-count-normalized (target sum of 10,000 per cell) and log-normalized. Data were zero-centered and scaled to unit variance for dimensionality reduction and clustering. Additionally, the scaled data were clipped to a maximum value of 10. Each sample was clustered separately using scanpy by performing (a) dimensionality reduction via PCA, (b) UMAP embedding, then (c) Leiden clustering on the scaled gene expression data. Only genes included in the panel as cell type markers were used for clustering. Clustering resolutions of 0.5 (with a minimum distance of 0.5), 0.75 (with a minimum distance of 0.3), and 1.5 (with a minimum distance of 0) were attempted to find the optimal resolution. A resolution of 1.5 was used for analysis.

### Ligand-receptor analysis

Ligand-receptor analysis was performed using stlearn (v1.1.5)^21^ in Python 3.10.18 on mucosal single-cell data. Xenium data were gridded into 8×8 micron spots. Based on connectomeDB2020^22^, 22 ligand-receptor pairs were identified. Co-expression was analyzed within a diffusion distance of 150 microns. A background distribution of 20,000 random gene pairs was used for significance testing. P-values were adjusted via Benjamini-Hochberg correction (FDR < 0.05). Ligand-receptor pairs required expression in at least two spots for testing.

### CRISPR-Cas9 mutagenesis in zebrafish

58 pmol CRISPR RNA (crRNA) (5’-CATTGGGTAAAACTACATG-3’), 58 pmol FP-labelled trans-crRNA (Sigma), and 6.75 pmol Cas9 protein (Sigma) were combined and allowed to complex for 30 min on ice before injection. Wild-type AB zebrafish embryos were injected with 4 nl of injection mix per embryo at the 1–4-cell stage. Embryos were screened for the presence of fluorescent guide RNA at 2 hr after fertilization. At 5 days post-fertilization (dpf), larvae were collected for gDNA extraction. A region of genomic DNA containing the intended CRISPR target sites was amplified by PCR (F primer: 5’-CTGCAGTGATGGATTCTTTGCT-3’; R primer: 5’- CAGCATGGTAAGTGTGTTTCG-3’). PCR products were purified using the Qiaquick PCR Purification Kit (Qiagen). The EnGen Mutation Detection Kit (NEB) was used to identify mosaicism for *csf2rb* mutations. Once T7 digestion indicated the presence of CRISPR/Cas9-induced mutations, injected fish were added to the system and raised to adulthood. At 3 months of age, CRISPR F0 fish were outcrossed against wild-type AB zebrafish, and pooled resultant larvae were screened for mutations as previously described. Clutches positive for mutations in *csf2rb* were added to the system and raised to adulthood. At 2–3 months of age, F1 fish were fin-clipped. PCR and gel electrophoresis were used to identify zebrafish heterozygous for a deletion mutation in *csf2rb*. gDNA from mutant heterozygotes was amplified using PCR and sequenced by Sanger sequencing (Genewiz). Fish that were predicted to carry the same mutation became founders for the mutant line and were in-crossed. Individual resultant embryos were sequenced with Sanger sequencing (Genewiz) to confirm the mutant sequence. The resulting mutation is a single base pair insertion in exon 3 (NC_007114.7:g.24346850_24346851insG). This results in a frameshift mutation predicted to yield a substitution of 14 amino acids followed by an early stop codon (NP_001107205.1:p.Val86GlyfsX15). These mutants were assigned the allele name ‘mss15’ by The Zebrafish Information Network (ZFIN).

### Chemical treatment of zebrafish

Single DSS treatment of zebrafish larvae was carried out as per published protocols^15^. 7 days post-fertilization zebrafish larvae were treated with 0.25% (w/v) colitis grade DSS (36,000–50,000 MW, MP Biomedical) for 24 hours with or without 350nM recombinant human (rh)GM-CSF (R&D Systems). Recovery conditions consisted of the removal of DSS and returning of zebrafish to egg water with or without 350nM rhGM-CSF for 5 hours.

### Lck-GFP^+^ cell isolation and scRNA-seq

Lck-GFP+ cells were isolated from larval zebrafish intestines using the MACSQuant Tyto Cell Sorter (Miltenyi Biotec). Cells were suspended in 1 mL PBS + 0.5% BSA, sorted at 4 mL/hr (<150 Pa), and diluted to 50 μL. Cells were counted (Nexcelom Auto 2000), and 40 μL of sorted cells were loaded into the 10X Genomics Chromium controller with the Single-Cell A Chip Kit. cDNA libraries were prepared using the Chromium Single-Cell 3′ Library, Gel Bead Kit v2.

### Larval zebrafish gut length measurement and neutral red quantification

Larval zebrafish intestines were measured and neutral red staining was performed as per published protocols^15,16^. The ratio of intestine length to body length was used to account for individual growth rate variations. Larval zebrafish were imaged using Nikon SMZ745 scope. The zebrafish gut was selected using ImageJ and average intensity of neutral red staining was measured.

### Larval zebrafish scRNA-seq

scRNA-seq on larval zebrafish intestines was performed according to previously published protocols^16^. N = 30 larval zebrafish intestines were dissected per condition. 10,000 cells were loaded onto the 10X Genomics Chromium controller with the Single-Cell A Chip Kit (10X Genomics). The cDNA libraries were generated using the Chromium Single-Cell 3′ Library, Gel_Bead_Kit_v2 (10X Genomics). Libraries were sequenced on Illumina Novaseq. The raw (unfiltered) output matrices were used for the clustering and downstream analysis in R package Seurat v4.0 (X). Data were filtered to include cells with 150 < features (unique genes) < 3500, and mitochondrial transcripts < 30%. Samples were individually normalized, and the 2000 most variable genes were identified for each sample. ‘FindIntegrationAnchors’ was used to integrate all zebrafish samples. The data were scaled and dimensionality reduction was performed. For integrations of zebrafish samples, the first 15 principal components were used to generate clustering, with a resolution of 0.8. Enriched genes for each cluster were identified using the ‘FindAllMarkers’ function. The default Wilcoxon Rank Sum test was used to determine significance, with a cutoff of Log_2_(fold change) = 0.25.

### Ingenuity pathway analysis on DEGs

Marker genes DGE was calculated for ILC3 versus ILC1 as described in the methods above. Differentially expressed genes, log-fold changes, and p values were imported into ingenuity pathway analysis (IPA) for all data sets (https://digitalinsights.qiagen.com/IPA) Core analysis was conducted on each data set using log-fold change values.

### Differential gene expression (DGE) analysis

DGE between clusters and samples was determined using the ‘FindMarkers’ function in Seurat v4.0^23^, with the Wilcoxon Rank Sum test for significance.

### Gene expression visualization

To visualize gene expression across ILC clusters and condition in a heatmap, the plot heatmap function was used from the R package Scillus (https://github.com/xmc811/Scillus).

### Single-cell velocity analysis

RNA velocity was analyzed across four conditions using Velocyto (v0.17) to generate loom files, which were merged with Seurat objects and processed in scVelo (v0.2.4) to model transcription dynamics^24^. Cell velocities indicated gene expression changes, and phase plots highlighted ILCs per treatment group.

### RNAscope in-situ hybridization

Fixation, cryosectioning, and immunofluorescence of larval zebrafish followed established protocols^25^. Larvae were fixed in 4% PFA overnight at 4°C, incubated in 30% sucrose, embedded in OCT, and stored at −80°C. Sections were mounted on VWR^®^ Premium Superfrost^®^ Plus slides. RNAscope ISH (ACD Bioscience) used four probe sets targeting csf2rb, il7r, rorc, ifng1.1, il22, tnfa, tbx21, il1b, and osm. Negative controls omitted probes. Sections were post-fixed, dehydrated, treated with Protease IV, and hybridized with diluted probes. Fluorophores identified C1-C4 targets. DAPI stained nuclei, and slides were mounted in EcoMount. Imaging (Zeiss 880, x20/x40) was standardized, with ≥5 zebrafish per condition. ImageJ was used for processing and quantification.

### Immunohistochemistry

Fixation, cryosectioning, and immunofluorescence of larval zebrafish were performed as described in the RNAscope in situ hybridization section. Cryosections (10 μm) were incubated overnight at 4°C with pSTAT5 (Tyr694) antibody (Invitrogen) at a dilution of 1:25 (2 μg), followed by incubation with Alexa Fluor 555-conjugated anti-mouse IgG (1:500; Invitrogen). Nuclei were counterstained with DAPI, and slides were mounted using EcoMount. Imaging was performed using a Zeiss 880 microscope at 40× magnification and standardized across samples, with at least 10 zebrafish per condition. ImageJ/Fiji was used for image processing and quantification.

Human tissue specimens were fixed in 10% formalin, paraffin-embedded, and sectioned at 3μm. Highplex IF was performed on the VENTANA-DISCOVERY-ULTRA (Roche Diagnostics) for automated processing. Adjacent sections were incubated with 1:6 CD14-PE (R&D) and DAPI or 1:6 GM-CSF-Alexa405, 1:30 pSTAT3-Alexa488, 1:30 pSTAT5-PE, and 1:30 pERK1/2-Alexa647 (Cell Signaling) for 1 hour at RT. Slides were mounted with Vectashield (Vector). Images were acquired at 300X using a Leica Stellaris 8 confocal microscope, merging 200–380 tiles per section. Pearson correlation coefficients were calculated using Coloc 2 (ImageJ/Fiji).

### Quantification and statistical analysis

Data points represent biological replicates, consisting of the mean of an individual patient or animal (as detailed in figure legends) ±SE. All statistical analyses were conducted using Prism (v9; GraphPad Software). The differences for means were tested for statistical significance with Mann–Whitney–Wilcoxon Test.

### Materials availability

The *csf2rb*^*mss15*^ allele information is available at The Zebrafish Information Network (https://zfin.org/ZDB-ALT-220708-1#summary). Requests for embryos can be made to Ling-shiang Chuang (ling-shiang.chuang@mssm.edu) and Jaime Chu (jaime.chu@mssm.edu).

## Results

### Differential myeloid cell growth factor abundance by inflammation and source-target mappings defined by spatial transcriptomics in ileal Crohn’s disease

**Figure 1.**
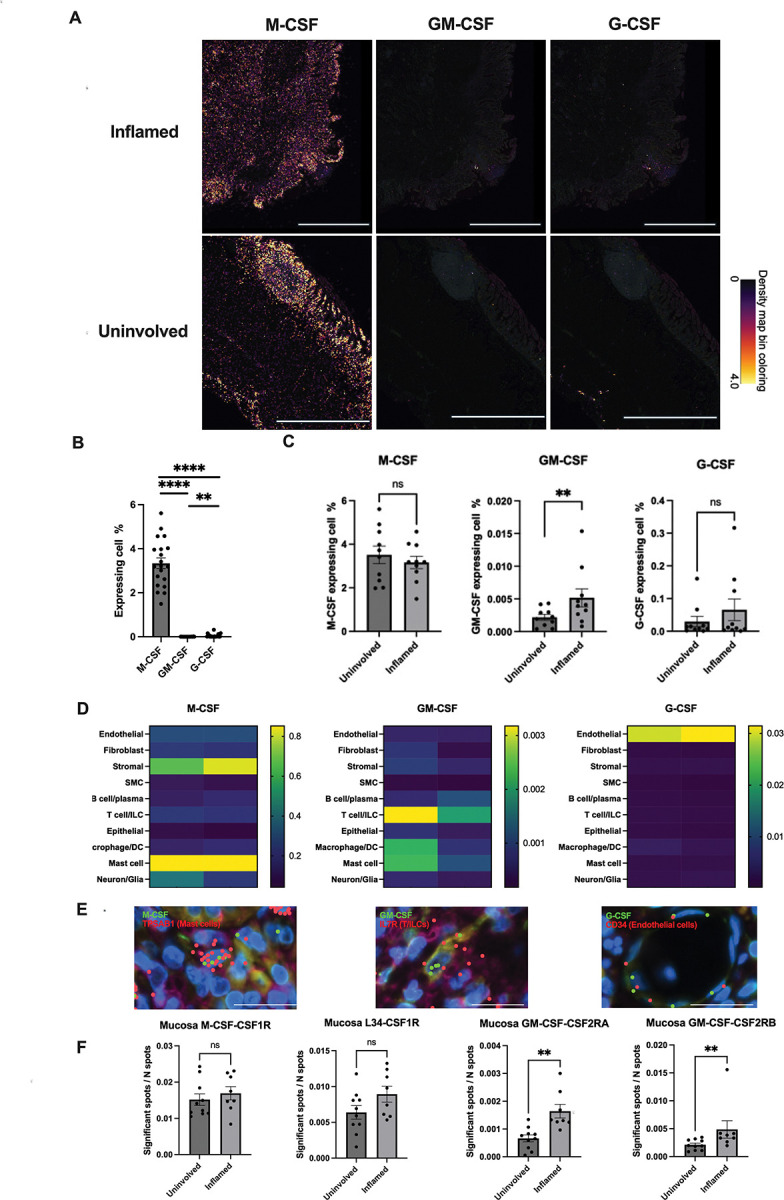
Elevated and localized GM-CSF expression in inflamed Crohn’s Disease ileums with stable M-CSF and G-CSF levels. **A.** Ileal Crohn’s disease tissue sections (inflamed and uninvolved regions) were profiled using 10X Xenium. Transcript density maps for M-CSF, GM-CSF, and G-CSF are color-coded: yellow (high), purple (intermediate), and black (none). Scale bar, 2000 μm. **B.** Bar graph quantifying the percentage of cells expressing each CSF per Xenium slide from 10 patients (10 inflamed and 10 uninvolved slides). **C.** Bar graph compares CSF expressions between inflamed and uninvolved regions (N = 10, **, P < 0.01). **D.** Heat map of CSF expression per cell cluster (yellow: high, green: medium, purple/black: low/none). **E.** Images of CSF with markers: TPSAB1 (mast cells), IL7R (T/ILC), and CD34 (endothelial cells). Scale bar, 20 μm. **F.** Bar graphs of ligand-receptor interactions in inflamed vs. uninvolved ileal sections. The significant Spot to N spot sample ratio was calculated as the number of ligand-receptor interactions in the same grid (Spot) relative to the total spots in the full mucosa area (10 uninvolved slides and 8 inflamed slides). **, P < 0.01.

We performed Xenium *In Situ* transcriptome profiling on paired inflamed and uninvolved ileal resection tissues from ten CD patients comparing M-CSF (CSF1), GM-CSF (CSF2) and G-CSF (CSF3). We observed markedly higher expression of M-CSF compared to GM-CSF and G-CSF (Figure 1A-B). underscoring its role in maintaining myeloid cell homeostasis in the gut^26,27^. Additionally, GM-CSF was uniquely enriched in inflamed vs. uninvolved regions across paired patient samples (Figure 1C), highlighting its importance specifically during inflammation.

Xenium datasets revealed expression of M-CSF predominantly in stromal and mast cells and G-CSF in endothelial cells, with minimal differences due to inflammation status (Figure 1D, **Figure S1**). In contrast, GM-CSF demonstrated the greatest per cell expression in lymphocytes (T cells/ILCs) in inflamed tissues, with more modest per cell expression in myeloid cells (macrophages, dendritic cells, and mast cells). The cellular sources of CSFs identified by Xenium spatial transcriptomics were validated using published single-cell RNA sequencing data from Martin *et al.* Consistent with our findings, CSF2RB, the β-chain of the GM-CSF receptor, was broadly expressed across multiple cell types, including endothelial cells, B/plasma cells, monocytes/macrophages, dendritic cells (DCs), and mast cells. In contrast, the co-receptor CSF2RA displayed a restricted expression pattern, being detected exclusively in monocytes/macrophages and dendritic cells, indicating that these populations are the principal targets of GM-CSF signaling within the intestinal microenvironment (**Figure S2**). Spatial analysis using stLearn revealed that the ratio of significant GM-CSF ligand–receptor interaction spots (GM-CSF with either CSF2RA or CSF2RB) to total spots was significantly increased in inflamed compared to uninvolved intestinal tissue sections. This pattern was distinct from that observed for M-CSF and its receptor interactions, which showed no comparable change between conditions (Figure 1F). The unique induction of GM-CSF and its receptors CSF2RB and CSF2RA in neighboring cells in inflammation compared to the other myeloid growth factors, combined with the genetic associations of CSF2RB (including a frameshift, loss-of-function allele^5,28^) warranted further investigation into the role of GM-CSF during intestinal inflammation.

### Human GM-CSF mitigates intestinal inflammation in zebrafish in a Csf2rb-dependent manner

To test the functional role of the GM-CSF pathway during intestinal inflammation *in vivo*, we generated a novel zebrafish knockout line for *csf2rb*, the zebrafish ortholog of human *CSF2RB*. To generate *csf2rb*-deficient animals, we injected zebrafish embryos with CRISPR-Cas9 complex comprised of CRISPR RNA targeting *csf2rb* exon 3, fluorescently-labeled trans-activating CRISPR RNA, and Cas9 protein. Fluorescent-positive embryos, indicating successful delivery of CRISPR-Cas9 (**Figure S3A**), acquired mutations in the target region as assessed by T7 endonuclease I digest. Sequencing analysis identified a single-nucleotide (guanine) insertion in exon 3 of *csf2rb*, which was predicted to result in a frameshift that induces an early stop codon, yielding a truncated form of Csf2rb consisting of 99 amino acids (**Figure S3B-C**).

**Figure 2.**
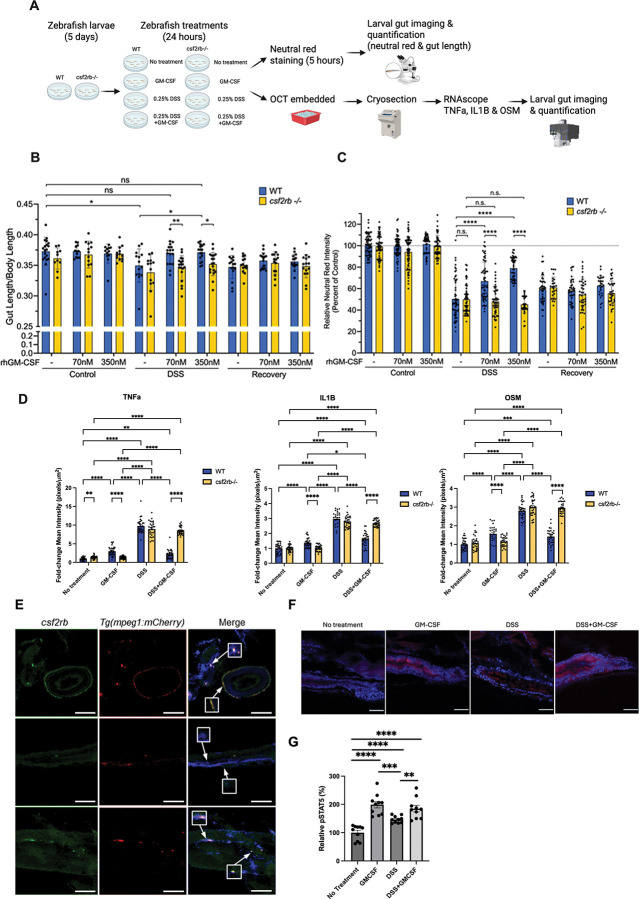
Human GM-CSF prevents injury in zebrafish DSS model of intestinal injury through Csf2rb signaling. **A.** Diagram of strategies on zebrafish treatments and data collections. **B.** Bar graph of intestinal length relative to total body length for WT (blue, N = 12–19/condition) and *csf2rb*^−/−^ (yellow, N = 10–19/condition) larval zebrafish treated with DSS and GM-CSF. *, P < 0.05, **, P < 0.01. **C.** Bar graph of neutral red staining in the zebrafish intestine relative to genotype-matched controls for WT (blue, N = 28–70/condition) and *csf2rb*^−/−^ (yellow, N = 25–68/condition) larval zebrafish treated with DSS and GM-CSF. ****, P < 0.0001. **D.** Quantification of RNA foci of RNAscope images for the proinflammatory cytokines *tnfα*, *il1b*, and *osm* in larval zebrafish intestines treated with DSS and human GM-CSF (*N* = 5, six images per larval gut). **, *P* < 0.01; ****, *P* < 0.0001. **E.** RNAscope images for *csf2rb* (green) with macrophage reporter (red), *Tg(mpeg1:mCherry)*, larval zebrafish. Colocalized regions (white) was shown in merge. Scale bar: 100 μm. **F.** Immunostaining of phospho-STAT5 (red) and nuclei (blue, DAPI) with wildtype larval zebrafish treated with DSS and GM-CSF. Scale bar: 50 μm. **G.** Quantification of relative intensity of phospho-STAT5 per larval zebrafish gut. N = 10 per treatment conditions. **, *P* < 0.01; ***, P>0.001; ****, *P* < 0.0001.

We induced intestinal injury with DSS and either co-treated with human GM-CSF or removed DSS after established inflammation and then treated with GM-CSF, respectively (Figure 2A). Co-treatment with GM-CSF significantly reduced intestinal injury as measured by partial rescue of lysosome-rich enterocyte uptake of neutral red, a proxy for assessing epithelial cell function^29,30^, and rescued gut length, with loss of Csf2rb completely abrogating rescue by GM-CSF (DSS) (Figure 2B-C). Furthermore, GM-CSF appeared to provide no benefit during mucosal healing (recovery) indicating that GM-CSF signaling exerts its protective effect during active injury and inflammation *in vivo*.

To determine how GM-CSF further modulated the immune signaling environment of the inflamed intestine, we assessed expression of key cytokines in inflammation by RNAscope. Expression of pro-inflammatory cytokines *tnfa*, *il1b*, and *osm* were all induced with DSS treatment regardless of genotype; however, GM-CSF significantly blunted this increase specifically in WT zebrafish (Figure 2D, **S4A**). Importantly, in WT zebrafish, GM-CSF alone did induce pro-inflammatory gene expression, albeit at much lower levels than DSS treatment. However, these inductions of proinflammatory cytokines were abrogated in *csf2rb*^−/−^ zebrafish (Figure 2D, **S4A**). To validate that *csf2rb* expression was conserved in zebrafish macrophages, we performed RNAscope to visualize *csf2rb* expression in *Tg(mpeg1:mCherry)* zebrafish larvae, a transgenic line that labels macrophages with mCherry. *csf2rb* demonstrated co-localization with mCherry^*+*^ macrophages in the intestine, midbody, and retina (Figure 2E, **S4B**), indicating that *csf2rb* expression is conserved in macrophages across zebrafish and human. To validate that human GM-CSF treatment can activate CSF2RB–pSTAT5 signaling in the zebrafish gut, pSTAT5 immunostaining was performed on WT zebrafish following GM-CSF and DSS treatment conditions. pSTAT5 levels were significantly increased with GM-CSF, DSS, and GM-CSF+DSS treatments compared to untreated controls, with the largest increase observed following exogenous GM-CSF administration (Figure 2F-G).

### ILCs with conserved gene expression profiles exist within the larval zebrafish gut

**Figure 3.**
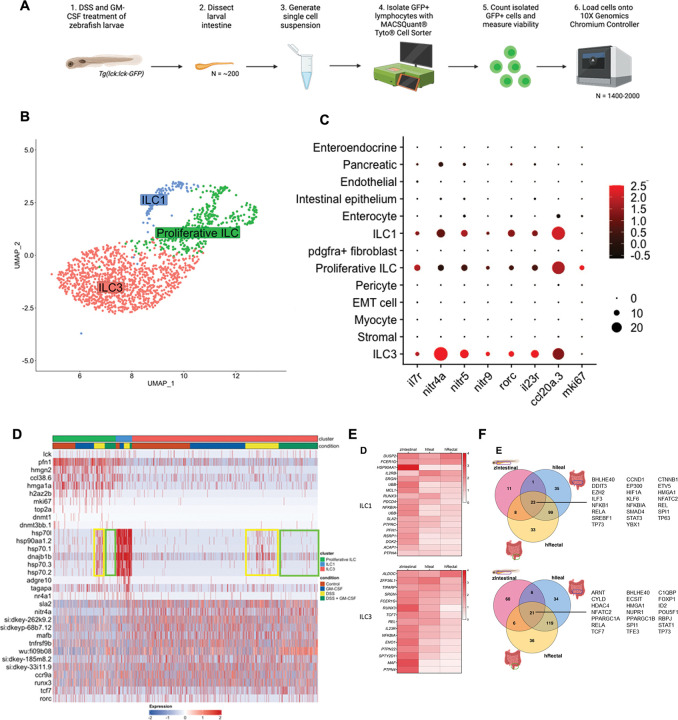
Single-cell transcriptomic profiling reveals conserved ILCs and marker signatures in the larval zebrafish intestine. **A.** Diagram of strategies on isolation and single-cell analysis of innate lymphoid cells (N = 200 larval guts/condition). **B.** UMAP of lck-positive ILCs from larva zebrafish intestine. **C.** Dotplot of conserved ILC3 markers (*rorc* and *il23r*), previously reported zebrafish ILC markers (*il7r, nitr4a*, *nitr5*, *nitr9, ccl20a.3*) and proliferation marker (*mki67*) in lymphocytes in zebrafish larvae scRNAseq data. **D.** Top differentially expressed genes for zebrafish proliferative ILCs (top bar, green), ILC1s (top bar, blue), and ILC3s (top bar, orange) in no treatment control (bottom bar, red), GM-CSF (bottom bar, blue), DSS (bottom bar, yellow) and DSS GM-CSF cotreatment (bottom bar, green). The yellow rectangle highlights the induction of ILC1 gene module by DSS treatment in proliferative ILCs and ILC3s, while the green rectangle indicates the reversal of the ILC1 gene module upon cotreatment with human GM-CSF. **E**. Heatmap of conserved ILC1 (top panel) and ILC3 (bottom panel) marker genes in zebrafish intestine, human ileum, and human rectum. **F.** Venn diagram of conserved transcriptional regulators predicted by IPA for ILC1s (top panel) and ILC3s (bottom panel) across human ileum and rectum, and larva zebrafish intestine.

Given that ILCs are potent expressors of GM-CSF in the inflamed human intestine and demonstrate dynamic population regulation, with reduced ILC3s and expanded ILC1s commonly found in inflamed CD patient tissues^4^, we reasoned that ILCs likely play an important role in GM-CSF-mediated protection against intestinal inflammation. Given that GM-CSF is largely produced by lymphocytes during intestinal inflammation, To this end, we characterized the lymphocyte populations present in the larval zebrafish intestine by dissecting intestines from Tg(lck:lck-GFP) zebrafish (N = ~200/condition), a reporter line labeling lymphocytes with GFP, isolated GFP^+^ cells, and performed scRNA-seq (Figure 3A). Analysis revealed three major clusters ILC clusters, identified by their expression of previously reported zebrafish ILC markers^17^ (*nitr4a*, *nitr5*, *nitr9*), and conserved ILC3 markers^10^ (*il7r*, *rorc*, *il23r*, *ccl20a.3*) in the largest ILC cluster (Figure 3B-C). We classified these three clusters as proliferative ILCs, ILC1s, and ILC3s based on the expression of characteristic genes (Figure 3C-D). The proliferative ILC population expressed proliferation markers such as *mki67* and *top2a* as well as the DNA methyltransferases *dnmt1*, *dnmt3bb.1* and DNA-associated components including *hmgn2*, *hmga1a*, and *h2az2b*. ILC1s were identified by high expression of a stress gene module including numerous heat shock protein family members such as *hsp70l*, *hsp90aa1.2*, and *dnajb1b* (Figure 3D). These observations are in line with human scRNA-seq data revealing an ILC1 population with similarly high expression of heat shock protein family members, a stress gene module, and high expression of *IFNG*, a key ILC1 effector cytokine^17,31^. ILC3s were identified by expression of known regulators of ILC3 identity *mafb*, *tcf7*, and *rorc*^32,33^ (Figure 3D).

To explore the similarity of zebrafish and human ILCs, we compared the marker gene profiles of ILC1s and ILC3s across our zebrafish intestinal and human ileal^19^ and rectal^34^ scRNA-seq datasets to identify conserved ILC marker genes. Zebrafish and human ILC1s expressed HSP90AA1, UBB, MCL1, and DOK2, while ILC3s expressed TCF7, IL23R, MAF, and PTPN22. RUNX3, critical for ILC1 and ILC3 identity, was enriched across both species (Figure 3E)^35^. Ingenuity Pathway Analysis revealed strong conservation of transcriptional regulators between zebrafish and human ILC1s, with ~75% overlap, including regulators of IL-2 and NF-kB pathways. For ILC3s, key transcriptional regulators, such as ARNT^36^, ID2^37^, RBPJ^38^, and TCF7^39^, were conserved. These findings highlight conserved ILC identity and function across species (Figure 3F).

### GM-CSF represses ILC1 activity and boosts the tissue repair function of ILC3s in intestinal inflammation

**Figure 4.**
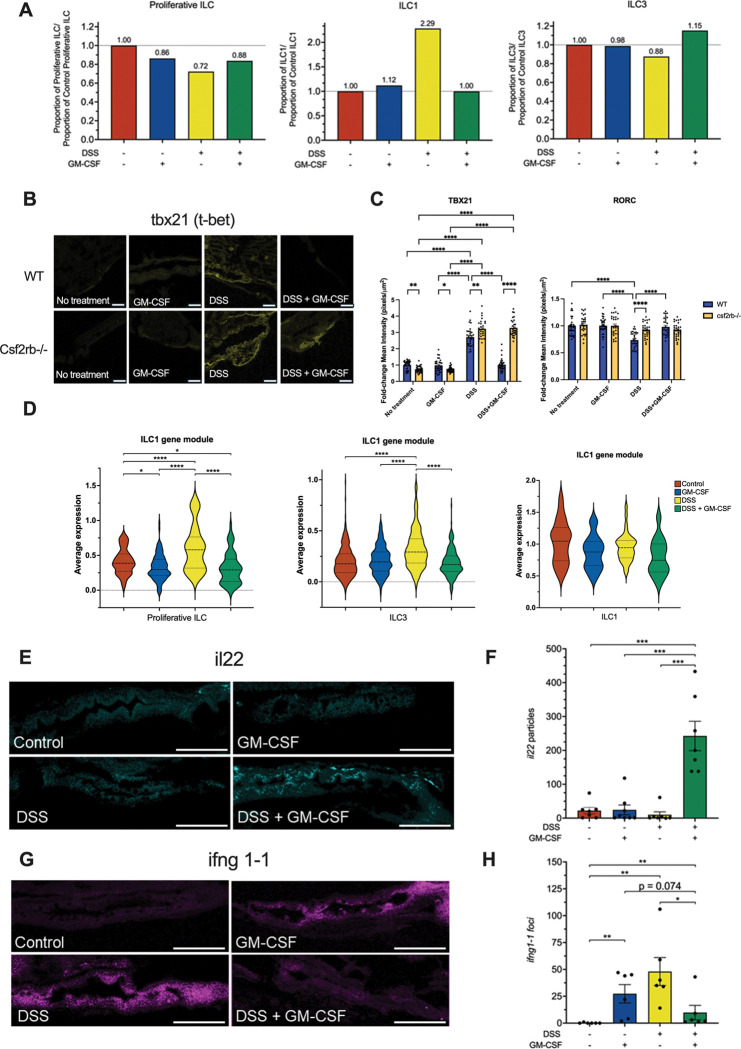
GM-CSF represses ILC1 activity and boosts the tissue repair function of ILC3s in intestinal inflammation. **A.** Zebrafish ILC proportions from scRNA-seq dataset. **B.** RNAscope images showing TBX21 probe staining in WT and csf2rb−/− zebrafish intestines treated with DSS and GM-CSF. Scale bar, 50 μm. **C.** Quantification of RNA foci for ILC markers tbx21 (ILC1) and rorc (ILC3) (N = 5 zebrafish, six images per gut). *, P < 0.05; **, P < 0.01; ****, P < 0.0001. **D.** Expression of ILC1 gene module in proliferative ILCs, ILC3s, and ILC1s under different treatments (red: control, blue: GM-CSF, yellow: DSS, green: DSS + GM-CSF). ****, P < 0.0001; *, P < 0.05. **E.** RNAscope images with il22 probe staining of zebrafish intestines treated with DSS and GM-CSF. Scale bar: 100 μm. **F.** Quantification of il22 RNA foci (N = 6–7, one image per gut). ***, P < 0.001. **G.** RNAscope images with ifng1–1 probe staining of zebrafish intestines treated with DSS and GM-CSF. Scale bar, 100 μm. **H.** Quantification of ifng1–1 RNA foci (N=6–7, one image per gut). **, P < 0.01; *, P < 0.05.

To assess ILC dynamics under intestinal inflammation and GM-CSF signaling, we compared ILC proportions across control, GM-CSF, DSS, and DSS + GM-CSF conditions in zebrafish intestines. This revealed a more than two-fold increase in ILC1s with DSS treatment compared to control conditions, which was rescued to control levels by co-treatment with GM-CSF (Figure 4A). DSS treatment also resulted in a slight decrease in proliferative ILCs and ILC3s, with the ILC3 decrease also being rescued by GM-CSF co-treatment (Figure 4A).

To validate these findings, we used RNAscope *in situ* hybridization^40^ on larval zebrafish tissues following DSS +/− GM-CSF treatment. Total intestinal ILC numbers appeared to be relatively stable across conditions as was demonstrated by similar levels of *il7r* (**Figure S4C-D**), a marker for ILCs^41^. To demonstrate that the impacts of GM-CSF on ILC1 and 3 population dynamics during intestinal inflammation are dependent on GM-CSF signaling, we analyzed expression of lineage-defining transcription factors *tbx21* and *rorc*, respectively. While DSS induced significant accumulation of *tbx21* expression regardless of genotype, GM-CSF co-treatment rescued this increase only in WT zebrafish (Figure 4B-C, **S4A**). Furthermore, DSS induced a decrease in *rorc* levels specifically in WT zebrafish that was also rescued by co-treatment of DSS (Figure 4C, right panel, **S4A)**. DSS-treated zebrafish larvae mirror CD patient ILC shifts, with GM-CSF via Csf2rb supporting ILC3s and limiting ILC1 expansion during inflammation.

Interestingly, proliferative ILCs and ILC3 also showed a marked increase in the expression of ILC1 marker genes specifically under conditions of gut inflammation (Figure 3D). To quantify this, we generated a module of ILC1 marker genes and calculated the average expression across single ILCs. We found that DSS treatment significantly induced ILC1 gene signatures in both proliferative ILC and ILC3 populations, which was completely abrogated by co-treatment with GM-CSF (Figure 4D). To further assess ILC function we evaluated expression of characteristic ILC cytokines by RNAscope. *Il22*, a characteristic cytokine of ILC3s that plays a key role in regulation of the intestinal barrier^42^, was specifically and highly induced by DSS and GM-CSF co-treatment, but not DSS or GM-CSF alone (Figure 4E-F, **S4D**). Conversely, the proinflammatory ILC1 cytokine, IFNG (*ifng1–1*), was significantly induced by DSS treatment and partially rescued by GM-CSF co-treatment (Figure 4G-H, **S4D**). These data further demonstrate that, during inflammation, GM-CSF plays a critical role in supporting ILC3 function while dampening ILC1 function.

### GM-CSF maintains native ILC identities during intestinal inflammation

**Figure 5.**
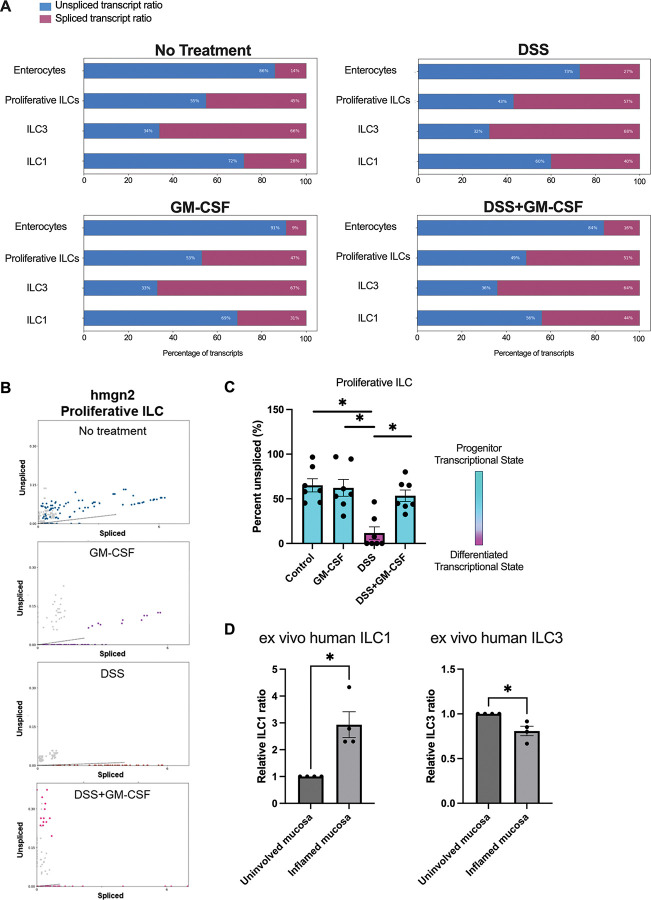
Plasticity of intestinal ILCs. **A.** Unspliced (red) to spliced transcript (blue) ratio of intestinal ILC and enterocytes in control (top left panel), GM-CSF (bottom left panel), DSS (top right panel) and DSS with GM-CSF co-treated (bottom right panel) larval zebrafish determined by single-cell velocity analysis. **B.** Unspliced to spliced transcript ratios of proliferative ILC marker gene, *hmgn2* in control (top row, blue punctation), GM-CSF (second row, purple punctation), DSS (third row, red punctation) and DSS with GM-CSF co-treated (bottom row, pink punctation) larval zebrafish (N = 15/data point). **C.** A bar graph of percent unspliced transcript on top seven differential express genes in proliferative ILC cluster indicates the progenitor or differentiated transcriptional states. *, P < 0.05. **D.** Quantification of relative ILC1 and ILC3 cell proportion in inflamed and uninvolved mucosa of human ileal tissue. *, P < 0.05.

Given that intestinal inflammation induced alterations to ILC identities, which were rescuable by GM-CSF, we performed RNA velocity analysis on our zebrafish ILC scRNA-seq dataset to better characterize ILC dynamics. Terminally differentiated cell types, such as enterocytes, demonstrated low levels of unspliced transcripts, indicative of low levels of active transcription to support homeostatic functions (Figure 5A). In contrast, proliferative ILCs, ILC1s, and ILC3s all demonstrated greater proportions of unspliced versus spliced transcripts (Figure 5A), highlighting that ILCs exist in a more active state, potentially indicating active replication and/or differentiation^43^.

The proliferative ILC population we identified possibly represents a population of ILCs actively undergoing cell cycling that may represent a more ‘progenitor-like’ population^31^. A marker of proliferative ILCs, *hmgn2*, has been demonstrated to play a critical role in maintaining progenitor populations across numerous tissues and cell types^44^. Analysis of the proportion of unspliced versus spliced *hmgn2* transcripts revealed that DSS-induced intestinal inflammation specifically reduced new *hmgn2* transcription (Figure 5B). When we quantified these proportions for a module of top proliferative ILC marker genes, we similarly found significantly fewer new (unspliced) transcripts with DSS treatment. GM-CSF co-treatment rescued transcription of proliferative ILC marker genes to control levels (Figure 5C, **S5A**). Taken together with the fact that DSS induced ILC1-like gene expression in proliferative ILCs, these data indicate that DSS may be driving ILCs to a more ILC1-like phenotype, but GM-CSF is sufficient to protect native ILC states.

ILCs are known to be particularly plastic in intestinal inflammation, with ILC3s often converting into ex-ILC3s that take on an ILC1-like phenotype^43,45^. Analyzing our Xenium data on ileal tissue from CD patients, we similarly found an expansion of ILC1s (*IL7R*^+^ *KLRB1*^+^ *TBX21*^+^) and contraction of ILC3s (*IL7R*^+^ *KLRB1*^+^ *RORC*^+^) in inflamed versus uninvolved tissue, consistent with previous reports^12,46^ (Figure 5D, **S5B**). Taken together, these data demonstrate that GM-CSF is sufficient to protect ILCs from alterations to their native identities driven by intestinal inflammation.

### pSTAT5^+^ CD14^+^ macrophages spatially correlate with GM-CSF in the inflamed intestine

**Figure 6.**
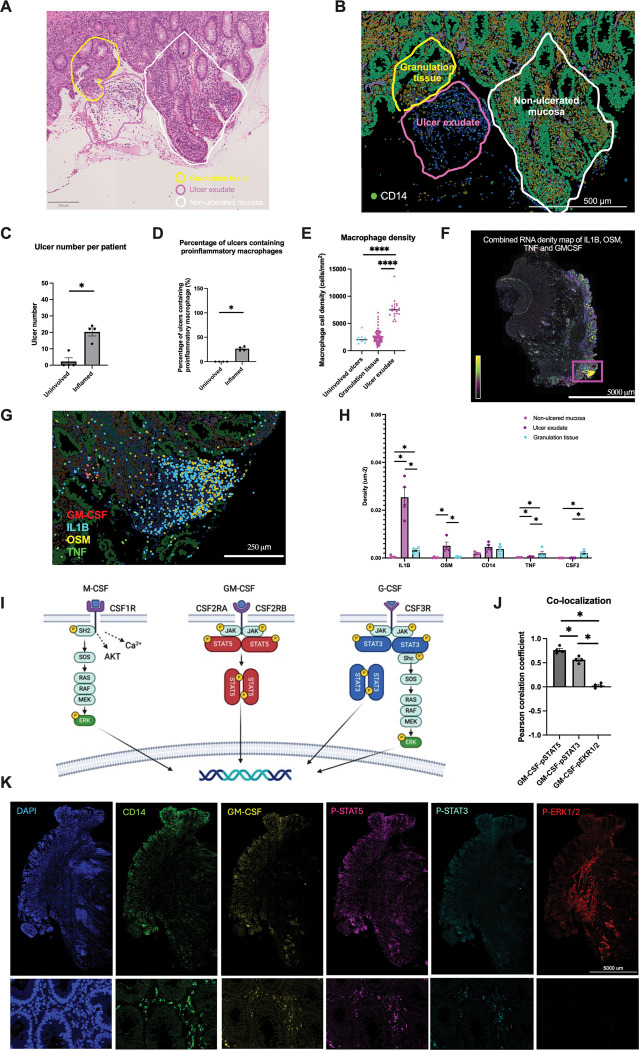
Spatial activation of phopho-STAT5 signaling pathway surrounding proinflammatory macrophage aggregates. **A.** H&E image of inflamed CD section with non-ulcerated mucosa (white), ulcer exudate (pink) and granulation (yellow). Scale bar, 200 μm. **B.** Xenium images of inflamed CD section with non-ulcerated mucosa (white), ulcer exudate (pink), and granulation tissue (yellow). Scale bar, 500 μm. **C-E.** Ulcer numbers, macrophage adjacency, and macrophage density quantifications. * P < 0.05, **** P < 0.0001. N = 4 patients with paired inflamed and uninvolved sections. **F.** GM-CSF and cytokine (IL1B, TNF, OSM) density map. Scale bar, 5000 μm. **G.** Expression map of GM-CSF (red), IL1B (light blue), OSM (yellow), and TNF (green) showing IL1B and OSM enrichment in macrophage aggregates at ulcer exudate. Scale bar, 250 μm. **H**. Bar graphs of cytokine and CD14 transcript density in non-ulcered mucosa, ulcer exudate, and granulation tissue. *, P < 0.05. N = 4 patients with paired inflamed and uninvolved sections. **I.** Schematic of M-CSF, GM-CSF and G-CSF signaling through pERK, pSTAT5, pSTAT3/pERK, respectively. **J.** Bar graph showing the Pearson correlation coefficient from averaging five macrophage aggregate regions per inflamed ileal section. **K.** Representative images of inflamed ileal tissue with co-immunostaining for DAPI, CD14, GM-CSF, p-STAT5, p-STAT3 and p-ERK1/2 (top panel). Scale bar, 5000 μm. Zoomed-in regions of macrophage aggregates show DAPI, CD14, GM-CSF, p-STAT5, p-STAT3 and p-ERK1/2 (bottom panel). Scale bar, 250 μm.

Intestinal ulceration, a common feature of CD inflammation^47^ characterized by disruption of the mucosal barrier, epithelial loss, and infiltration of inflammatory cells such as macrophages and neutrophils, is composed of ulcer exudate, made up of inflammatory cells, and granulation tissue, which includes newly formed blood vessels^48^ (Figure 6A). While M-CSF exhibited widespread expression, GM-CSF and G-CSF were specifically expressed within ulcer granulation tissue in the inflamed ileum (Figure 1A). While proinflammatory macrophages were largely restrict to the ulcer exudate, macrophages were occasionally present in the granulation tissue (Figure 6B–F, **S6A-B**). Interestingly, while inflammatory cytokines IL-1β and OSM were predominantly expressed within macrophage-rich exudate, TNF and GM-CSF were mainly localized to the granulation tissue (Figure 6G–H, **S6C**). These findings suggest that ulcer exudate and granulation tissue represent distinct inflammatory microenvironments with specialized cytokine signaling profiles.

Given the spatially restricted nature of GM-CSF, we aimed to explore if its downstream signaling occurred in a similar manner. STAT5 phosphorylation (pSTAT5) occurs downstream of GM-CSF engagement and multimerization of its receptors CSF2RA and CSF2RB. Unlike GM-CSF, M-CSF and G-CSF signal through ERK and STAT3 phosphorylation, respectively (Figure 6I). To assess CSF signaling spatially, we performed immunofluorescent staining for GM-CSF, pSTAT5, pSTAT3, and pERK1/2 on inflamed ileal sections, with DAPI and CD14 staining on adjacent sections. By quantifying the Pearson Correlation Coefficient at a single cell resolution between GM-CSF and the phosphorylated signaling proteins, we observed a strong and significant colocalization between GM-CSF and pSTAT5 compared to pSTAT3 and pERK1/2 (Figure 6J). GM-CSF and pSTAT5 were localized to macrophage aggregates labeled with CD14, consistent with previous transcriptomic findings (Figure 6K and **S6D**). In conclusion, local GM-CSF promotes STAT5 phosphorylation within macrophage aggregates, potentially contributing to maintenance of tissue homeostasis in response to inflammation.

## Discussion

**Figure 7.**
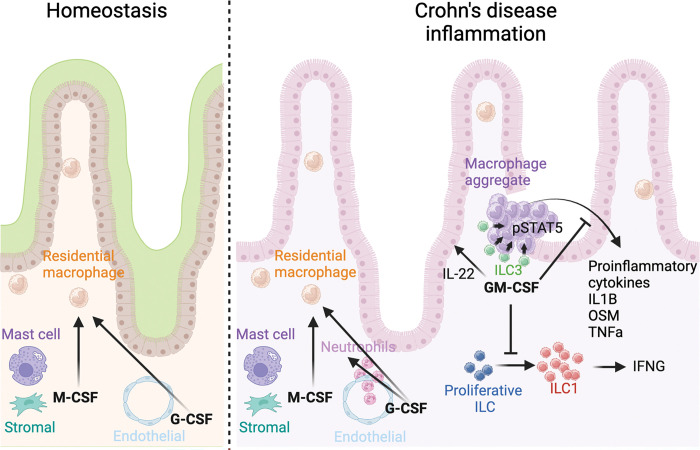
GM-CSF in ulceration modulates ILC state and proinflammatory cytokines. **A.** Model: M-CSF, GM-CSF and G-CSF in homeostasis and Crohn’s disease inflammation.

GM-CSF, M-CSF, and G-CSF direct myeloid cell differentiation, and myeloid cells are key drivers of inflammation and tissue damage in IBD^49^. Thus, clarifying the distinct roles of CSFs in CD pathogenesis remains essential. Genome-wide association studies (GWAS) have significantly advanced our understanding of inflammatory bowel disease (IBD). Our previous research identified a single nucleotide polymorphism (SNP) in *CSF2RB* as the second most prevalent frameshift SNP associated with CD in the Ashkenazi Jewish population, following *NOD2*^5^. Additionally, a recent study by Liu et al. in *Nature Genetics*^50^ reported a strong association between *CSF2RB* and CD in the East Asian population, with an exceptional P-value of 10^−20^, ranking third after *IL23R* and *ADO*. These findings underscore the critical role of *CSF2RB* in both Jewish and Asian populations. Given that loss of GM-CSF and its receptors, expressed largely by myeloid cells, increases CD risk^5,28^, GM-CSF likely plays a protective role in intestinal inflammation. In this report, we identify GM-CSF signaling niches comprised of innate lymphoid and myeloid cells that are induced specifically in granulation tissue of ulcers within inflamed ileal tissue from CD patients. We further show that GM-CSF mitigates intestinal injury and proinflammatory gene induction (*il1b*, *osm*, *tnfa* and *ifng1–1)*, prevents expansion of inflammatory ILC1s, supports ILC3 maintenance and induction of pro-epithelial repair *il22*, and sustains ILC progenitor-like states during acute intestinal inflammation *in vivo* (Figure 7).

We leveraged novel Xenium *In Situ* single-cell spatial transcriptomics on inflamed (and uninvolved) ileal tissues from CD patients to generate datasets with improved resolution. This fine-scale spatial resolution allowed us to identify that while M-CSF and G-CSF transcripts are abundant in the intestinal mucosa, GM-CSF is uniquely responsive to inflammation and is spatially localized within ulcerated regions, i.e. areas of active inflammatory disease. In contrast, M-CSF is evenly distributed, and G-CSF is mainly confined to blood vessels. This spatial distinction highlights a unique role for GM-CSF in CD inflammation. Interestingly, rather than being expressed in the ulcer exudate comprised of myeloid cells highly expressing proinflammatory *IL1B* and *OSM*, GM-CSF was expressed within the granulation tissue, a site of tissue repair after inflammatory injury. These observations may point to point to a role for GM-CSF in controlling inflammation to promote tissue healing as opposed to propagating inflammatory responses. Furthermore, even at low abundance, cytokines like GM-CSF can exert strong effects localized within disease-specific microenvironments. Given that ILC3s are the major producers of GM-CSF in the gut, as previously reported and confirmed by our data^51^, this highlights myeloid-lymphoid spatial interactions within ulcers as central axes in CD pathogenesis.

To characterize GM-CSF signaling during intestinal inflammation *in vivo*, we generated the first zebrafish *csf2rb* knockout line (*csf2rb*^*mss15*^). While *CSF2RB* expression is conserved in myeloid cells between humans and zebrafish, orthologs for *CSF2RA* and *CSF2* remain unidentified—even with support from the Zebrafish Information Network (ZFIN)—likely due to low cross-species gene sequence conservation. Many zebrafish cytokines share conserved protein structures and functional similarities with their mammalian counterparts despite low sequence homology. Our data show that recombinant human GM-CSF rescues DSS-induced intestinal injury in zebrafish, an effect that is abolished in *csf2rb* knockout animals, confirming conserved GM-CSF signaling via Csf2rb. Signaling conservation was further validated by a significant increase in pSTAT5 levels in the zebrafish gut following human GM-CSF stimulation. Additionally, transcriptomic profiling demonstrate that zebrafish macrophages recapitulate key phenotypic and functional traits of human intestinal macrophages, including the expression of genes associated with inflammatory signaling and tissue remodeling. These findings establish zebrafish as a robust model for studying disease-relevant myeloid dynamics in intestinal injury.

ILCs are dynamically regulated by inflammation with ILC1s expanding and ILC3s contracting in IBD^4,46^. Furthermore, the level of serum anti-GM-CSF autoantibodies correlates with increased ileal ILC1s in CD patients^4^, highlighting a potential role for GM-CSF in the regulation of ILCs. Intestinal ILCs have been previously profiled in human intestinal tissue, as well as one manuscript identifying ILC-like cells in the adult zebrafish intestine^17,52,53^. To study the potential for GM-CSF to modulate ILCs, we identified and generated, to our knowledge, the first transcriptional characterization of ILCs present in the larval zebrafish intestine. We found that larval zebrafish intestinal ILCs demonstrate considerable conservation of key genes and transcription factors known to be important for ILC function and identity with human intestinal ILCs. ILC3s also represented the dominant ILC population in the larval zebrafish gut, demonstrating additional similarity to human ILC populations. Consistent with this, scRNAseq data from zebrafish models indicate that ILC3s are approximately 11.2 times more abundant than ILC1s (Figure 3F), with total ILC numbers remaining stable across conditions (Supplemental Figure 9, *il7r*). Given the twofold increase in ILC1s during DSS injury, the slight decrease observed in ILC3s is likely attributable to their initially much higher baseline numbers relative to ILC1s.

Further, we demonstrated that human GM-CSF regulates ILC number and function during intestinal inflammation in our larval zebrafish model. While this model lacks adaptive immunity^54^ and does not fully recapitulate the immune environment in CD, it provides the unique opportunity to interrogate myeloid-ILC crosstalk independent from adaptive lymphocytes with similar identities and functions, namely T helper cells. Furthermore, this avoids the need for ablation of immune compartments by *Rag1/2*^−/−^ and antibody-depletion based methods, commonly employed in mice, that disrupt the native immune system and can also alter disease-relevant responses. However, to further strengthen the physiological relevance of our findings, future studies should examine human intestinal ILCs isolated from patient-derived tissue or intestinal explants. This would enable direct assessment of GM-CSF-mediated signaling in a human immune context and enhance the translational value of our zebrafish model.

Additionally, we identify a population of proliferative ILCs that may represent an ILC progenitor-like cell type. In humans, ILC progenitors are abundant in the fetal intestine and decreased in the adult intestine^52,53^. Our scVelo analysis demonstrated that DSS injury caused zebrafish proliferative ILCs to lose new transcription of marker gene, *hmgn2*, highlighting a push towards a more ILC1-like phenotype in response to intestinal inflammation. *HMGN2* is known to play a role in proliferation, and is enriched in many progenitor cells^44^, further pointing to these proliferative ILCs as potential progenitor-like cells. Importantly, GM-CSF seems to reverse the push of ILCs to an ILC1-like phenotype, further implicating it as a regulator of ILCs during intestinal inflammation. Due to the lack of CSF2RA/B on ILCs, this effect is likely mediated indirectly through monocytes—potentially via retinoic acid signaling to ILC3s, as described in previous studies^4,5^.

GM-CSF signaling is enriched during inflammation. Our spatial transcriptomic analysis reveals that GM-CSF, and proinflammatory cytokines TNF, IL1B, and OSM, are expressed in distinct niches within ulcers in CD. Previous studies have shown that the *CSF2RB* frameshift mutation that causes increased risk for CD results in decreased pSTAT5 in response to GM-CSF in macrophages^5^. Furthermore, mutation of *STAT5A* and *STAT5B* correlates with increased risk for CD^2,55^ and mice deficient in STAT5 tetramerization are hypersusceptible to DSS colitis^56^. We found that pSTAT5, but not pERK, colocalizes with GM-CSF in these aggregates, indicative of active GM-CSF. Although *CSF3* and pSTAT3 also appear in the aggregates, pSTAT3 demonstrated significantly less colocalization with GM-CSF. Together, these findings highlight the role of GM-CSF-induced pSTAT5 signaling within monocyte aggregates in Crohn’s disease, underscoring its distinct spatial activation from M-CSF and G-CSF, and further supporting its unique contribution to restoring intestinal homeostasis. While GM-CSF transcripts were localized within ulcer granulation, GM-CSF protein was detected in both granulation tissue and ulcer exudate. This is likely due to the fact that GM-CSF is secreted and able to diffuse from the producing cell. Therefore, spatial detection of the protein may not directly correlate to RNA localization.

Importantly, our study goes beyond descriptive mapping by delineating the functional consequences of GM-CSF signaling through combined spatial molecular profiling and interventional *in vivo* modeling. Numerous studies have highlighted proinflammatory roles for GM-CSF in intestinal inflammation^7,51,57^; however, using novel Xenium In Situ characterization of inflamed ileal tissue from CD patients alongside zebrafish modeling, we define an anti-inflammatory role for GM-CSF mediated through ILC orchestration. By maintaining the equilibrium between immune cell populations, GM-CSF ensures that inflammatory responses are kept in check, preventing excessive tissue damage and dysregulated immune responses that characterize CD. This nuanced insight underscores the critical role of GM-CSF as a guardian during intestinal inflammation, highlighting its potential as a therapeutic target for restoring balance in the immune microenvironment and mitigating inflammation in Crohn’s disease. Our mechanistic investigations establish causal relationships between spatial CSF expression, immune cell dynamics, and tissue pathology, representing a significant advance in understanding the functional implications of spatial cytokine regulation in mucosal inflammation.

### Ethics Statement for Human Participant and Animals

Patients were identified by screening surgical programs at Mount Sinai Hospital. Protocols were reviewed and approved by the Institutional Review Board (IRB) at the Icahn School of Medicine at Mount Sinai (HSM#14–00210). Detailed information regarding the participants and resection samples can be found in **Supplemental table 1-2**.

All zebrafish experimental procedures were approved (protocol #: IACUC-2015–0050 & IPROTO202100000045) by Mount Sinai Institutional Animal Care and Use Committee (IACUC) in accordance with NIH guidelines for the humane treatment of animals. Adult fish were maintained on a 14:10 light/dark cycle at 28°C. Fertilized embryos collected following natural spawning were cultured at 28.5°C in egg water (0.6 g/l Instant Ocean Sea Salt; Blacksburg) containing methylene blue (0.002 g/l). AB WT, *csf2rb*^*mss15*^, *Tg(lck:lck-GFP)*^*cz2Tg* 58^, *Tg(mpeg1:mCherry)*^*gl23* 59^ zebrafish strain were used.

## Data Availability

Single-cell RNA-seq data have been deposited at GEO and are publicly available as of the date of publication. All original code has been deposited and is publicly available as of the date of publication.
